# Concurrent epirubicin and trastuzumab use increases complete pathological response rate without additional cardiotoxicity in patients with human epidermal growth factor receptor 2‐positive early breast cancer: A meta‐regression analysis

**DOI:** 10.1002/cam4.70005

**Published:** 2024-07-24

**Authors:** Ming‐Han Yang, Chiun‐Sheng Huang, Dwan‐Ying Chang, Fu‐Chang Hu, Shu‐Min Huang, Po‐Hsiang Huang, I‐Chun Chen, Tom Wei‐Wu Chen, Ching‐Hung Lin, Yen‐Shen Lu

**Affiliations:** ^1^ Department of Oncology National Taiwan University Hospital Taipei Taiwan; ^2^ Department of Surgery National Taiwan University Taipei Taiwan; ^3^ College of Medicine National Taiwan University Taipei Taiwan; ^4^ Graduate Institute of Clinical Medicine and School of Nursing, College of Medicine, National Taiwan University Taipei Taiwan; ^5^ Statistical Consulting Clinic, International‐Harvard (I‐H) Statistical Consulting Company Taipei Taiwan; ^6^ Department of Oncology National Taiwan University Cancer Center Taipei Taiwan; ^7^ Graduate Institute of Oncology, College of Medicine National Taiwan University Taipei Taiwan

**Keywords:** cardiotoxicity, early breast cancer, epirubicin, human epidermal growth factor receptor 2, trastuzumab

## Abstract

**Background:**

Due to cardiotoxicity concerns, the concurrent use of epirubicin and trastuzumab has not been fully studied. This study aimed to examine the cardiotoxicity and pathological complete response (pCR) rate associated with the concurrent regimens in patients with human epidermal growth factor receptor 2 (HER2)‐positive early breast cancer (EBC).

**Methods:**

We conducted a systematic search for relevant literature in the NCBI/PubMed, the Cochrane database, and international conference abstracts for phase II or III randomized controlled trials between January 1, 2000, and February 28, 2021, focusing on the concurrent regimens in patients with HER2‐positive EBC. To compare the risk of cardiotoxicity and the odds of the pCR rate, we performed linear meta‐regression analyses to investigate the effects of multiple covariates.

**Results:**

We analyzed 7 neoadjuvant trials involving the concurrent use of epirubicin and trastuzumab with 1797 patients. The median cumulative dose of epirubicin used was 300 mg/m^2^, with a total of 96 reported adverse cardiac events. The concurrent regimens did not result in a significant increase in cardiotoxicity compared to nonconcurrent regimens (risk ratio [RR] = 1.18, 95% confidence interval [CI] = 0.68–2.05). Compared with nonconcurrent or non‐anthracycline‐containing regimens, concurrent regimens were associated with a significant increase in the pCR rate (odds ratio = 1.48, 95% CI = 1.04–2.12). The linear fixed‐effects meta‐regression analysis indicated that in trials including more patients with hormone receptor‐positive EBC, the RR of cardiotoxicity significantly increased with concurrent regimens, and the pCR rate became less significant.

**Conclusions:**

The combination of trastuzumab and a low dose of epirubicin positively impacted the pCR rate without a significant increase in cardiotoxicity. We recommend exploring concurrent regimens for HR‐negative, HER2‐positive tumors to enhance pCR rates, with caution advised for HR‐positive tumors due to potential cardiotoxicity.

## Graphical abstract

Despite the emergence of multiple anti‐HER2 regimens, particularly anthracycline‐free neoadjuvant or adjuvant treatments, patients with HER2‐positive breast cancer continue to confront the reality of recurrence, metastasis, or disease progression. Our study showed the combination of trastuzumab and epirubicin had a positive impact on the pathological complete response rate without a significant increase in cardiotoxicity. Hopefully, this safety data illuminates a novel perspective for this specific patient cohort.

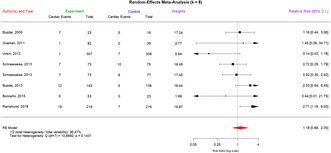

## 1 INTRODUCTION

In 2021, breast cancer overtook lung cancer as the most common form of cancer among women globally, representing 12% of all new annual cancer cases worldwide.^1^ Anthracyclines are some of the most effective chemotherapy drugs for treating breast cancer patients, especially those with human epidermal growth factor receptor 2 (HER2)‐positive breast cancer.^2^ In recent decades, trastuzumab, a monoclonal antibody targeting HER2, has substantially increased the survival rate of patients with HER2‐positive breast cancer.^3^, ^4^, ^5^, ^6^, ^7^, ^8^, ^9^, ^10^ However, the concurrent use of trastuzumab and anthracyclines is limited due to its potential to cause additive cardiotoxicity, which may not only increase mortality but also adversely affect the quality of life of breast cancer survivors.^11^, ^12^, ^13^, ^14^, ^15^, ^16^, ^17^, ^18^, ^19^, ^20^, ^21^, ^22^ The risk of heart failure persists as a lifelong concern, especially for young cancer survivors.

Balancing the antitumor efficacy of anthracyclines against their potential for cardiotoxicity presents a clinical challenge. At a cumulative doxorubicin dose of 400 mg/m^2^, 5% of the patients developed congestive heart failure; this proportion increased to 26% at 550 mg/m^2^.^15^ Efforts have been made to solve this problem, including the identification of anthracycline derivatives with less cardiotoxicity^12^ such as liposomal doxorubicin and epirubicin; the use of cardioprotective agents^19^; and the exploration of alternative treatments without anthracycline.^23^ Epirubicin is considered as an anthracycline with less cardiotoxicity. The probabilities of developing congestive heart failure are estimated to be approximately 0.9% and 3.3% at cumulative epirubicin doses of 550 and 900 mg/m^2^, respectively, both of which are considerably lower than the corresponding probability for doxorubicin.^11^, ^18^

This meta‐regression analysis aimed to compare the outcomes of concurrent epirubicin and trastuzumab treatment with or without pertuzumab (called concurrent regimens) with those of sequential epirubicin/trastuzumab treatment and non‐anthracycline treatment (called nonconcurrent regimens) before surgery, with a specific focus on cardiotoxicity and the pathological complete response (pCR) rate. There are several novel contributions to the existing body of research. Unlike previous studies, which have predominantly focused on the efficacy of the concurrent use of anthracyclines and trastuzumab,^24^, ^25^, ^26^, ^27^, ^28^, ^29^, ^30^, ^31^, ^32^ our analysis presented the safety issue, mainly cardiotoxicity, by using meta‐regression models, thus providing a more holistic understanding of these combination regimen. In addition, we used the generalized additive models (GAMs) to identify the appropriate cut‐off points for continuous covariates. This distinction not only enhances the depth of our understanding but also paves the way for future research in the combination use of epirubicin and trastuzumab.

## 2 MATERIALS AND METHODS

### 2.1 Search strategy

Our meta‐analysis followed the guidelines of the Preferred Reporting Items for Systematic Reviews and Meta‐Analyses (PRISMA) Statement. Two authors, Ming‐Han Yang (MH Yang) and Yen‐Shen Lu (YS Lu), independently conducted a comprehensive search of medical literature databases, including NCBI/PubMed, the Cochrane database, and international conference abstracts, covering the period from January 1, 2000 to February 28, 2021. The search terms used were “HER2‐positive breast cancer,” “neoadjuvant,” “adjuvant,” “trastuzumab,” and “anthracycline,” which were combined using the Boolean logic (Table S1).

### 2.2 Study selection

**FIGURE 1:**
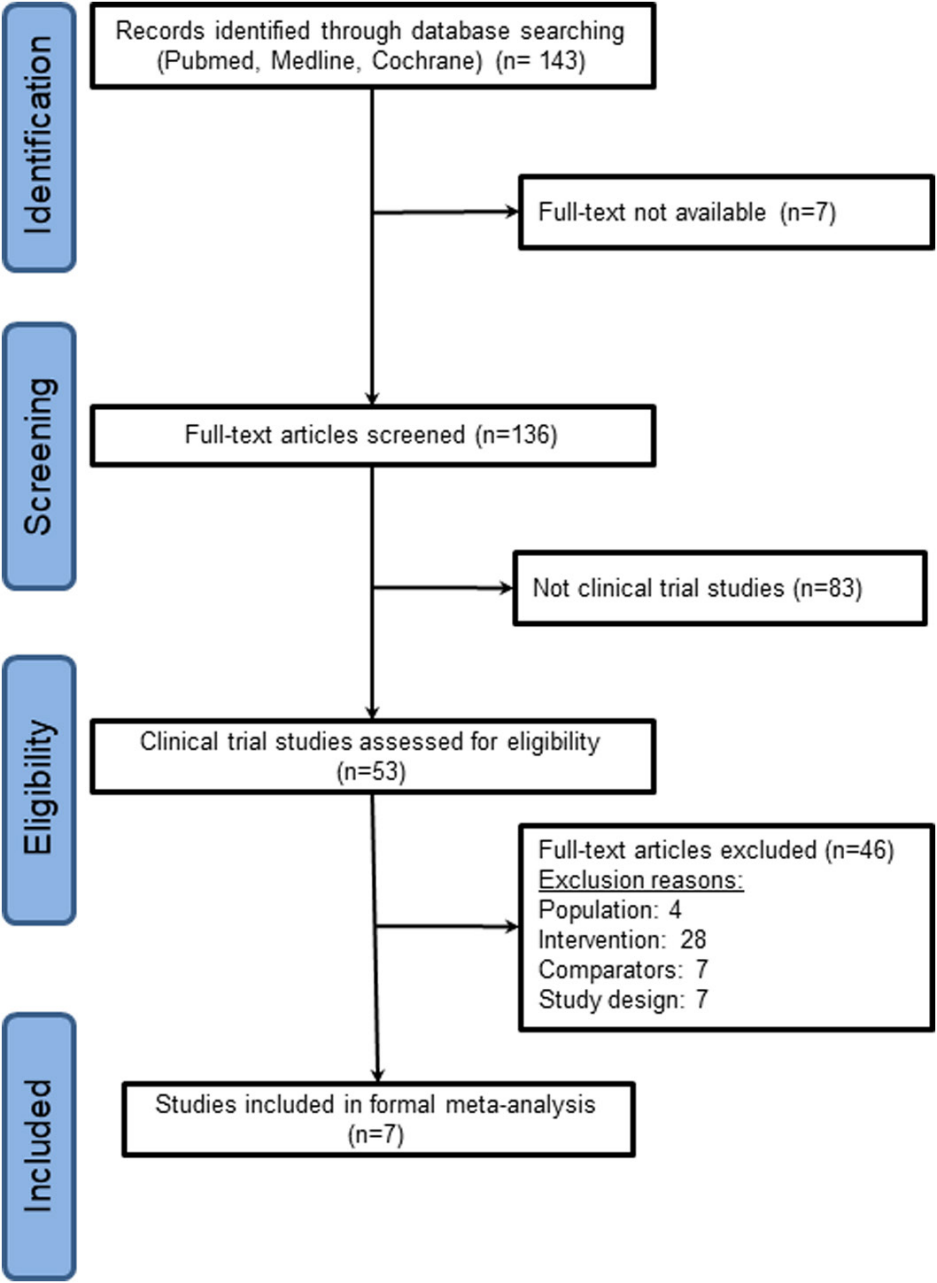
The Preferred Reporting Items for Systematic Reviews and Meta‐Analyses flow diagram of systematic literature search and trial selection process.

Two authors (MH Yang and YS Lu) independently reviewed the titles and abstracts of all retrieved studies to identify the relevant ones. The inclusion criteria are described as follows: (1) Phase II or III randomized controlled trials focusing on neoadjuvant or adjuvant treatment for HER2‐positive breast cancer, (2) concurrent use of epirubicin and trastuzumab, (3) reporting both cardiac events and pCR rates, and (4) publication in English. For studies that met these criteria, the authors obtained the full reports or contacted the study authors by email to obtain the necessary data. Subsequently, the two aforementioned authors independently assessed the eligibility of the retrieved studies. Phase I studies, case reports, studies without data on cardiotoxicity, or pCR rates, those that included metastases, and those with nonrandomized designs were excluded (Figure 1). Any disagreements in the assessments between MH Yang and YS Lu were resolved through discussion with another investigator Ching‐Hung Lin. The Cochrane tool for randomized trials was used to examine the risk of bias concerning the randomization process, deviation from the intended intervention, missing outcome data, outcome measurement, and selective reporting (Table S2).^33^

### 2.3 Data extraction and definition of cardiotoxicity

MH Yang and YS Lu independently extracted essential information from the seven selected trials to compile the meta‐data. They used a predefined form that included fields such as trial name, first author, publication year, journal, patient number treated, follow‐up duration, treatment regimens, patient characteristics (e.g., age, hormone receptor [HR] positivity, and echocardiography follow‐up schedule), all cardiac event rates, and pCR rates (Table 1).

**TABLE 1:** Characteristics of the 7 randomized clinical trials categorized into 8 groups.

Study name	Concurrent arm (con) versus nonconcurrent (non‐C) arm (N)^a^	Hormone receptor positive/total (%)	Cumulative dose of epirubicin (mg/m^2^)	T > 2 cm (%)	Node + (%)	UCG schedule	Definition of adverse cardiac events (ACE)	ACE rate	pCR rate
Buzdar MDACC 2005^24^	Con: TH(4) → FECH(4) Non‐C: T(4) → FEC(4)	24/42 (57.14%)	300 300	91.3% 89.47%	56.52% 63.16%	Baseline, Q3M until the end of FEC	LVEF decrease >10%	30.43% 26.32%	66% 25%
CHER‐LOB^34^, ^35^	Con: wT(L)H(12) → FEC(L)H(4) Non‐C: wTL(12) → FECL(4)	73/121 (60.33%)	300 300	100% 100%	NR NR	Baseline, Q12W, completion	LVSD (CTCAE 3.0) > gr 1	1.22% 0%	37% 26%
GeparQuinto GBG 44^36^, ^37^	Con: ECH(4) → DH(4) Non‐C: ECL(4) → DL(4)	341/615 (55.45%)	360 360	64.17% 65.58%	68.08% 67.53%	Baseline, Q4W, completion	Heart failure (CTCAE 4.03)	0.33% 2.27%	30% 23%
TRYPHAENA^28^, ^31^, ^b^ ^,^ ^c^	Con: FECPH(3) → DPH(3) Non‐C: FEC(3) → DPH(3)	74/148 (50.00%)	300 300	100% 100%	73.97% 65.33%	Baseline, Q6W, completion, Q6M for 2 years, and then QY for 2 years	All‐grade LVSD (CTCAE 3.0)	9.59% 13.33%	51% 28%
TRYPHAENA^28^, ^31^, ^b^ ^,^ ^c^	Con: FECPH(3) → DPH(3) Non‐C: DCarboPH(6)	79/150 (52.67%)	300 0	100% 100%	73.97% 68.83%	Baseline, Q6W, completion, Q6M for 2 years, and then QY for 2 years	All‐grade LVSD (CTCAE 3.0)	9.59% 10.39%	51% 40%
ACOSOG Z1041^27^, ^38^	Con: wTH(12) → FECH(4) Non‐C: FEC(4) → wTH(12)	168/280 (60.00%)	300 300	92.96% 94.20%	63.38% 64.49%	Baseline, Q12W, Q24W	LVSD (CTCAE 3.0)	8.45% 3.62%	51% 51%
EORTC 10054^29^	Con: DH(3) → FECH(3) Non‐C: DL(3) → FECL(3)	42/76 (55.26%)	300 300	100% 95.65%	67.92% 69.57%	Baseline, Q4W, completion	Absolute drop ≥15% in LVEF from baseline	0% 0%	51% 35%
TRAIN‐2^32^, ^39^, ^c^	Con: FECPH(3) → TCarboPH(6) Non‐C: TCarboPH(9)	255/438 (58.22%)	270 0	NR NR	62.56% 65.30%	Baseline, Q3M	LVEF decline of 10% or more AND LVEF <50%	8.68% 3.20%	64% 64%

Abbreviations: Con, concurrent arm; LVEF, left ventricular ejection fraction; LVSD, left ventricular systolic dysfunction; non‐C, nonconcurrent arm; NR, not reported; Q12W, every 12 weeks; Q3M, every 3 months; QY, annually; UCG, echocardiography.

^a^ Regimen abbreviations: T(H) paclitaxel (+ herceptin), FEC(H) fluorouracil + epirubicin + cyclophosphamide (+ herceptin), wT(L)H weekly paclitaxel + herceptin (+ lapatinib), D(H) docetaxel (+ herceptin), FECPH fluorouracil + epirubicin + cyclophosphamide + pertuzumab + herceptin, DPH docetaxel + pertuzumab + herceptin, DCarboPH docetaxel + carboplatin + pertuzumab + herceptin, TCarboPH paclitaxel + carboplatin + pertuzumab + herceptin.

^b^ TRYPHAENA was divided into two groups: concurrent arm versus nonconcurrent arm and concurrent arm versus non‐anthracycline arm.

^c^ The control arm of TRYPHAENA and TRAIN‐2 were anthracycline‐free regimens, which were considered of less cardiotoxicity.

Inconsistent definitions of cardiotoxicity used in the selected clinical trials have led to substantial variations in the estimated incidence of this condition (Table S3). Employing a composite endpoint of adverse cardiac events aimed to summarize the collective results from every study with various definitions of cardiotoxicity pre‐defined by different studies to increase the number of events and statistical power. However, its utilization can occasionally complicate result interpretation and subsequent clinical applications. Therefore, we adopted the most inclusive definition of adverse cardiac events. This endpoint included a decrease in left ventricular ejection fraction (LVEF) that exceeded 10% with LVEF below 50%, any grade of congestive heart failure, and any grade of left ventricular systolic dysfunction. In cases where a study was reported multiple times, we relied on the publication that focused primarily on cardiotoxicity, e.g., ASCO 2020 abstract for TRAIN‐2.^40^ The cumulative risk of trastuzumab‐associated cardiotoxicity increases for up to 2 years before stabilizing at around 3 years.^17^, ^41^ Therefore, we established a 2‐year observation period to assess the cumulative risk of cardiotoxicity. If a study lacked data at 2 years, we retrieved safety information from the nearest available time point.

### 2.4 Statistical analysis

Meta‐analyses and meta‐regression analyses were performed using the ‘metafor’ package, version 3.8‐1 (August 27, 2022), of the statistical software R, version 4.2.1 (R Foundation for Statistical Computing, Vienna, Austria). A two‐sided *p* value of ≤0.05 was considered statistically significant unless otherwise specified. The primary and secondary outcomes were cardiotoxicity and pCR rate, respectively. To examine the cardiotoxicity and evaluate the total pCR rate of concurrent regimens involving epirubicin and trastuzumab, we defined the natural logarithm of the estimated individual‐study risk ratio, log(RR), for cardiotoxicity, and the natural logarithm of the estimated individual‐study odds ratio, log(OR), for the total pCR rate as the effect measures. The weighted average of the individual‐study log(RR) for cardiotoxicity and log(OR) for the total pCR rate as the pooled effect size was calculated using the Mantel–Haenszel method. The results of random‐effects meta‐analyses with the Knapp and Hartung adjustment (for *k* < 30) were presented in two forest plots that displayed individual‐study effect sizes and the pooled effect sizes as the RR/OR with the corresponding 95% confidence intervals (CI). We evaluated heterogeneity between the selected studies with the chi‐squared *Q*‐test and the *I* ^2^ statistic, where the *p* value of the *Q*‐test <0.15 or *I* ^2^ >50% indicated significant heterogeneity.^42^ If significant heterogeneity was detected, we fitted a fixed‐effects linear meta‐regression model of log(RR) or log(OR) to the collected data using the weighted least squares method to find relevant covariates contributing to the observed heterogeneity. Moreover, if residual heterogeneity remained statistically significant, we added random effects to account for the unknown sources of residual heterogeneity to fit mixed‐effects linear meta‐regression models of log(RR) and log(OR). An RR = 1 indicated no difference between the treatments, an RR <1 indicated that the concurrent regimen of epirubicin and trastuzumab was associated with relatively low cardiotoxicity, and an RR >1 indicated that the control regimen was better in this regard. In addition, an OR >1 indicated that the concurrent regimen of epirubicin and trastuzumab was associated with a higher pCR rate.

Moreover, we applied the model‐fitting techniques for (1) variable selection, (2) goodness‐of‐fit (GOF) assessment, and (3) regression diagnostics in our meta‐regression analysis. At first, our stepwise variable selection procedure, including interactions between the forward and backward steps, was performed to identify the candidate final linear meta‐regression model for both cardiotoxicity and the pCR rate. We set the significance levels for entry and stay to 0.15 to be conservative. Then, we manually identified the best final linear meta‐regression model by removing the covariates with a *p* value >0.05 one at a time, aided by substantive knowledge, until all meta‐regression coefficients were significantly different from 0.

We fitted simple and multiple GAMs to draw GAM plots to determine continuous covariates' nonlinear effects and then to find the cut‐off value(s) for categorizing them, if necessary, in running the stepwise variable selection procedure. In practice, we used the vgam() function (with the default values of smoothing parameters) of the VGAM package, version 1.1‐7 (July 6, 2022),^42^, ^43^, ^44^ to fit the GAMs for our continuous responses in R, and then we used the plotvgam() function of the VGAM package to draw GAM plots in R to visualize continuous covariates' linear or nonlinear effects.

Next, we computed the coefficient of determination, *R* ^2^, which was the squared Pearson correlation between the observed and predicted responses from all included observations, to help us assess the GOF of the fitted linear meta‐regression model.

Finally, we examined publication bias, performed residual analysis, identified influential studies, and checked multicollinearity with the available statistical tools of regression diagnostics to discover any problems in the meta‐regression model or data. The potential publication bias was examined by drawing the funnel plot of the residuals from the fitted linear meta‐regression model, and then the asymmetry of the funnel plot was detected by Egger's regression test using the regtest() function of the metafor package. In the fitted linear meta‐regression model, if we found variance inflating factor (VIF) ≥ 0 in continuous covariates or VIF ≥2.5 in categorical covariates, the multicollinearity problem occurred among some of the covariates.

## 3 RESULTS

### 3.1 Systematic review of the literature

In this systematic review of the literature, we initially identified 290 articles and excluded 147 duplicates. Figure 1 presents the PRISMA diagram for our literature review. Seven studies were excluded due to the inaccessibility of the full text, and an additional 83 articles did not meet the criteria for clinical trials. Subsequently, we examined the eligibility of 53 randomized controlled trials. Among these trials, seven met the eligibility criteria and were included in the primary meta‐analysis; the baseline characteristics are summarized in Table 1. The seven trials were conducted in the neoadjuvant setting for a total of 1797 patients with early HER2‐positive breast cancer. Among these patients, 899 received concurrent regimens, while the remaining 898 received nonconcurrent regimens, including non‐anthracycline regimens. Of the seven trials, one was a multinational trial,^31^ two were conducted in the United States,^24^, ^27^, ^38^ and four were conducted in European countries^26^, ^29^, ^32^, ^34^, ^35^, ^36^, ^37^, ^39^, ^40^ (i.e., Germany, Italy, and the Netherlands). The median age of the 1797 patients was 49.5 years (range: 47–52). Approximately 56%–73% of the patients were node‐positive, 46%–65% had HR‐positive tumors, 34%–64% had grade 3 tumors, and 64%–100% had tumors measuring ≥2 cm in size. The median cumulative dose of epirubicin administered was 300 mg/m^2^ (range: 270–360 mg/m^2^). The median follow‐up duration was 6.44 months (range: 5.52–61). According to the Cochrane tool for assessing the risk of bias, the overall risk of bias in the included 7 trials was low (Table S2).

### 3.2 No difference in cardiotoxicity between concurrent and nonconcurrent uses of epirubicin and trastuzumab

The definitions of cardiotoxicity varied among the seven trials, and we aggregated these definitions under the composite term of ACE (Table 1). In our sensitivity analysis, which was performed to compare concurrent regimens with nonconcurrent regimens, we used a fixed‐effects model and observed no significant difference between these two types of regimens (risk ratio [RR] = 1.26, 95% CI = 0.84–1.89, *p* = 0.2593). Substantial heterogeneity was observed, although the level of heterogeneity was not statistically significant (chi‐squared *Q*‐test *x* ^2^ = 10.8992, *p =* 0.1431, *I* ^2^ = 35.77%). To account for this heterogeneity, we performed a random‐effects meta‐analysis with the Knapp and Hartung adjustment to compare concurrent regimens with nonconcurrent regimens (*I* ^2^ = 36.47%, *p =* 0.1431) and determined no significant difference between these two types of regimens (RR = 1.18, 95% CI = 0.68–2.05; *p* = 0.5650). Two studies took non‐anthracycline regimens as controls.^31^, ^32^ The results of a linear fixed‐effects meta‐regression analysis comparing concurrent regimens with non‐anthracycline regimens still revealed no significant differences in cardiotoxicity between these two types of regimens (RR = 1.63, *p* = 0.4130).

**FIGURE 2:**
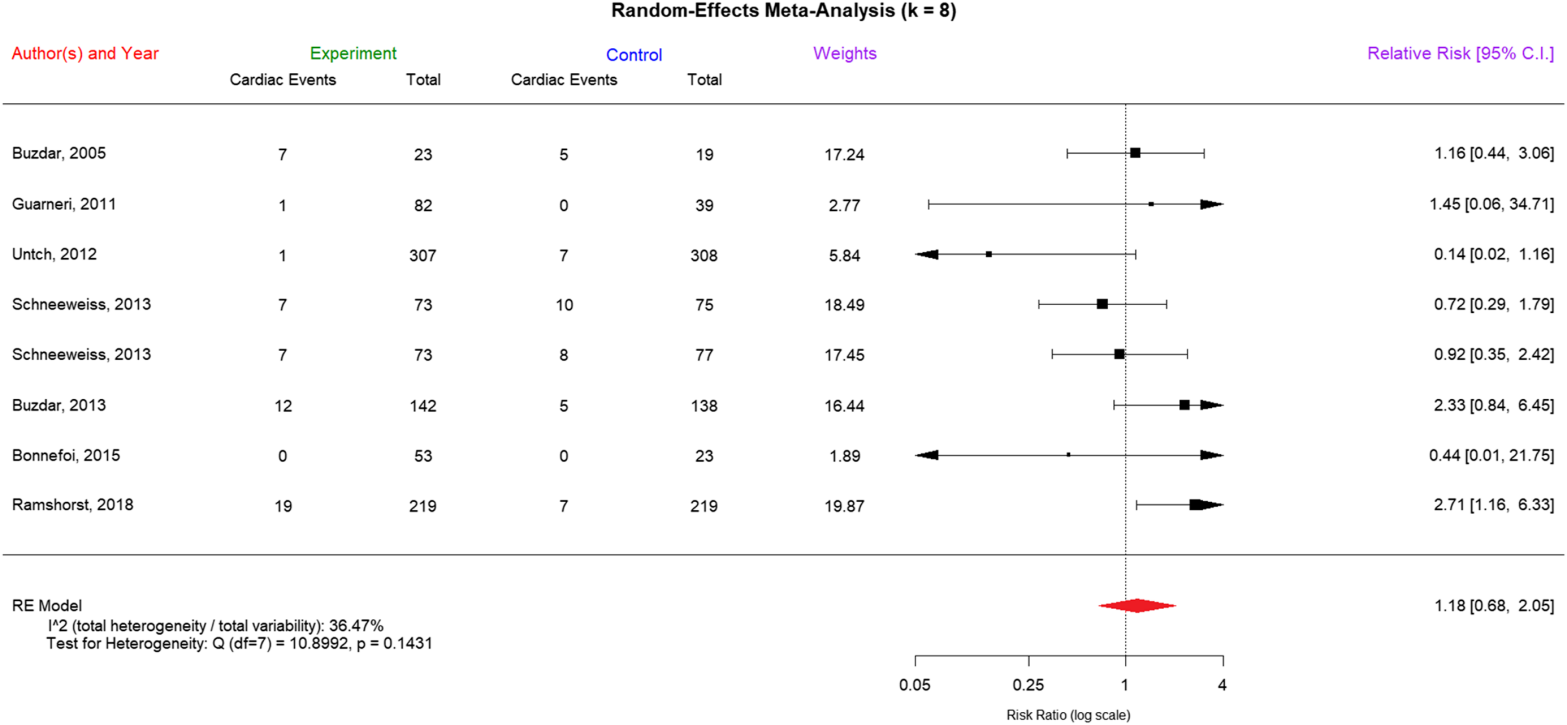
The forest plot of cardiotoxicity. A random‐effects meta‐analysis of the cardiotoxicity in concurrent epirubicin–trastuzumab regimens and nonconcurrent regimens was shown here. “Experiment” refers to the concurrent epirubicin–trastuzumab regimens and “Control” refers to the nonconcurrent regimens. An RR = 1 indicated no difference between the treatments, an RR <1 indicated that the concurrent regimen of epirubicin and trastuzumab was associated with relatively low cardiotoxicity, and an RR >1 indicated that the control regimen was better in this regard.

In the forest plot of Figure 2, we listed the results of the random‐effects meta‐analysis of cardiotoxicity in concurrent epirubicin–trastuzumab regimens and nonconcurrent regimens. Next, in Table 2, we present the results of the linear fixed‐effects meta‐regression analysis of log(RR) of cardiotoxicity in concurrent and nonconcurrent regimens with the aid of substantive knowledge, although there was no significant heterogeneity among the included trials (*I* ^2^ = 36.47% <50%). After adjusting for the effects of other covariates, we found that trials with HR(+) participants >57.20% would have a 1.0875 larger mean value of log(RR) of cardiotoxicity (95% CI: 0.2595–1.9154, *p* = 0.0100), but trials with control arm containing lapatinib would have a 1.3250 smaller mean value of log(RR) of cardiotoxicity (95% CI: −2.9775 − 0.3275, *p* = 0.1161), where the latter with borderline statistical significance was retained in the linear fixed‐effects meta‐regression model due to its potential importance in clinical practice. A GAM plot illustrated a nonlinear relationship between the proportion of patients with HR‐positive status and the log(RR) of cardiotoxicity, including an estimated cut‐off value of 57.20% (Figure S1A). Specifically, the mean value of log(RR) of cardiotoxicity in trials with HR(+) participants ≤57.20% and without lapatinib in the control arm was −0.1205 (95% CI: −0.6619 − 0.4208, *p* = 0.6625 >0.05). In contrast, the mean value of log(RR) of cardiotoxicity in trials with HR(+) participants >57.20% and without lapatinib in the control arm would be (−0.1205) + 1.0875 = 0.967, indicating that the RR of cardiotoxicity increased to exp (0.967) = 2.6300 >1.0. The multicollinearity problem was not detected by examining the value of VIF. The residual heterogeneity was not sufficiently large (test for residual heterogeneity: *QE* (*df* = 5) = 1.0755, *p* = 0.9563 >0.15; *I* ^2^ = 0.00% < 50%), so a linear mixed‐effects meta‐regression model was not needed. The *R* ^2^ = 0.8121 indicated that the linear fixed‐effects meta‐regression model fitted the meta‐data well.

**TABLE 2:** Multivariate analyses of cardiotoxicity and pathological complete response rate (pCR) by fitting two linear fixed‐effects meta‐regression models with the modern stepwise variable selection method^a^.

Covariate	Regression coefficient estimate	Estimated standard error	*p* value^c^	95% confidence interval
Cardiotoxicity (log RR)q				
Intercept	−0.1205	0.2762	0.6625	−0.6619−0.4208
Trials with HR(+) participants >57.20%	1.0875	0.4225	**0.0100**	**0.2595–1.9154**
Trials with a control arm containing lapatinib	−1.3250	0.8431	0.1161	−2.9775−0.3275
Fixed‐effects meta‐regression model (*QE* = 1.0755, df = 5, *p* = 0.9563; *I* ^2^ = 0.00% < 50%, *R* ^2^ = 0.8121)^b^				
pCR (log OR)				
Intercept	0.5582	0.1376	**<0.0001**	**0.2886–0.8278**
Trials with HR(+) participants >58.02%	−0.4911	0.1994	**0.0138**	**−0.8819 to −0.1003**
Fixed‐effects meta‐regression model (*QE* = 6.2820, df = 6, *p* = 0.3924; *I* ^2^ = 4.49% < 50%, *R* ^2^ = 0.3812)^b^				

^a^ A multivariable linear meta‐regression analysis of 7 randomized clinical trials with 8 observations was conducted using the escalc() and rma() functions in the ‘metafor’ package, version 3.8‐1 (2022‐08‐27), of the statistical software R, version 4.2.1 (R Foundation for Statistical Computing, Vienna, Austria), with the modern stepwise variable selection procedure, to fit the meta‐regression models of the log(hazard ratio) of cardiotoxicity and the log(odds ratio) of the pathological complete response (pCR) rate, respectively, where “log” was the natural logarithm.

^b^ The coefficient of determination, *R* ^2^, was the squared Pearson correlation between the observed and predicted responses from all included observations, which helped us assess the GOF of the fitted linear meta‐regression model.

^c^ The boldfaced *p* values reached statistical significance at α = 0.05.

### 3.3 Increased pCR rate with concurrent use of epirubicin and trastuzumab compared to nonconcurrent use

**FIGURE 3:**
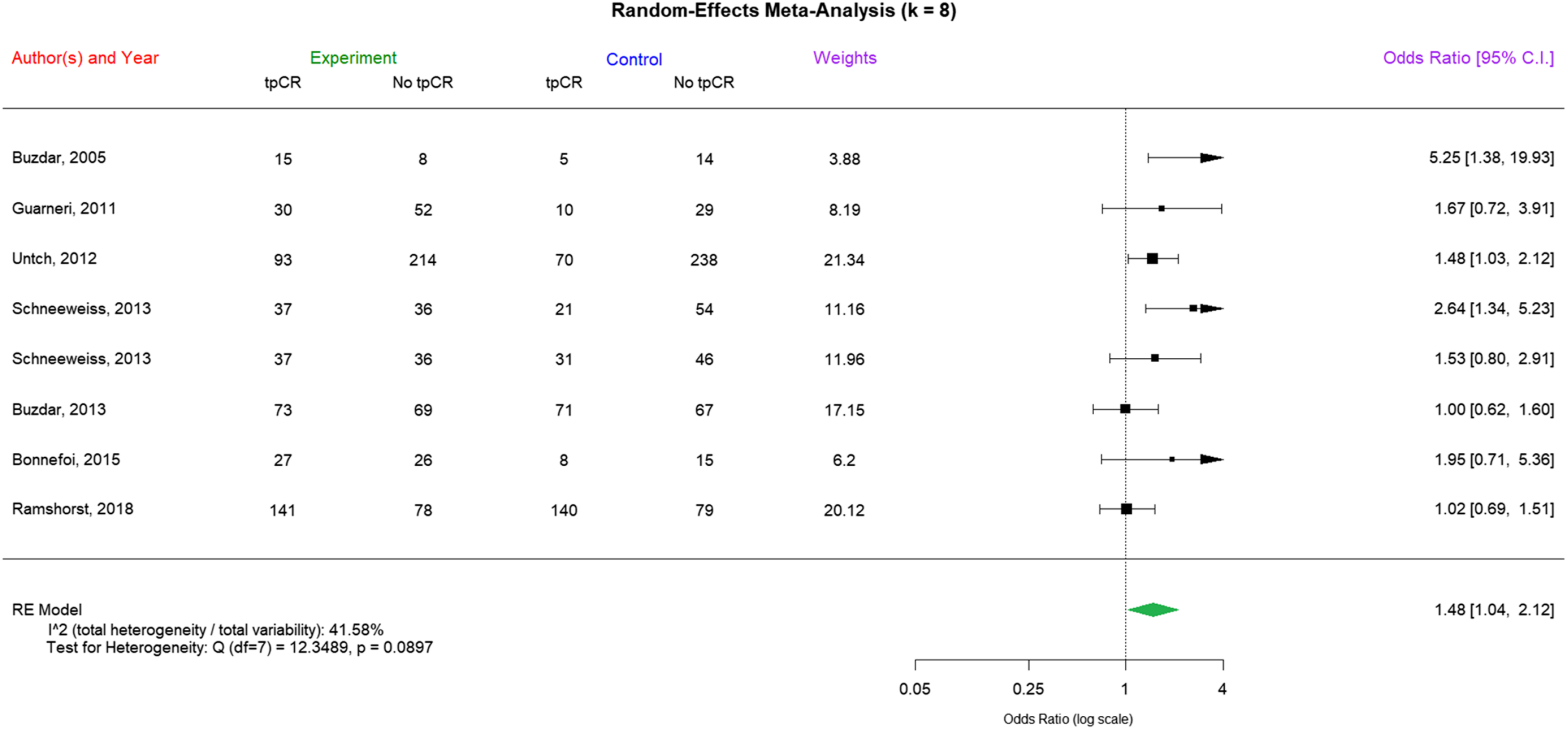
The forest plot of pathological complete response (pCR) rates. A random‐effects meta‐analysis of the pCR rate in concurrent epirubicin–trastuzumab regimens and nonconcurrent regimens was shown here. “Experiment” refers to the concurrent epirubicin–trastuzumab regimens and “Control” refers to the nonconcurrent regimens. An OR >1 indicated that the concurrent regimen of epirubicin and trastuzumab was associated with a higher pCR rate.

Similarly, in the forest plot of Figure 3, we listed the results of the random‐effects meta‐analysis of the pCR rate in concurrent epirubicin–trastuzumab regimens and nonconcurrent regimens. Next, in Table 2, we present the results of the linear fixed‐effects meta‐regression analysis of log(OR) of the pCR rate in concurrent and nonconcurrent regimens with the aid of substantive knowledge, although there was no significant heterogeneity among the included trials (*I* ^2^ = 41.58% < 50%). After adjusting for the effects of other covariates, we found that trials with HR(+) participants >58.02% would have a 0.4911 smaller mean value of log(OR) of the pCR rate (95% CI: −0.8819 to −0.1003, *p* = 0.0138). A GAM plot illustrated a nonlinear relationship between the proportion of patients with HR‐positive status and the log(OR) of the pCR rate, including an estimated cut‐off value of 58.02% (Figure S1B). Specifically, the mean value of log(OR) of the pCR rate in trials with HR(+) participants ≤58.02% was 0.5582 (95% CI: 0.2886–0.8278, *p* < 0.0001). In contrast, the mean value of log(OR) of the pCR rate in trials with HR(+) participants >58.02% would be 0.5582 + (−0.4911) = 0.0671, indicating that the OR of the pCR rate decreased to exp (0.0671) = 1.0694 = 1.0. The multicollinearity problem was not detected by examining the value of VIF. The residual heterogeneity was not sufficiently large (test for residual heterogeneity: *QE* (*df* = 6) = 6.2820, *p* = 0.3924 >0.15; *I* ^2^ = 4.49% < 50%), so a linear mixed‐effects meta‐regression model was not needed. The *R* ^2^ = 0.3812 indicated that the linear fixed‐effects meta‐regression model fitted the meta‐data just fairly.

### 3.4 Publication bias and sensitivity analysis

As shown in the two funnel plots in Figure S2A,B of the Supplementary Appendix, publication bias was not a concern because there was no significant evidence of asymmetry in these two funnel plots (test for funnel plot asymmetry *p* = 0.4891 and 0.0997 for cardiotoxicity and pCR rate, respectively). Sensitivity analysis revealed that none of the studies substantially affected the combined results of this meta‐analysis.

## 4 DISCUSSION

The introduction of early breast cancer screening and advanced neoadjuvant and adjuvant anti‐HER2 treatments have significantly improved the pCR rates and reduced mortality rates in patients with HER2‐positive breast cancer. However, systemic treatments, including anthracyclines and trastuzumab, have been associated with an increased risk of cardiotoxicity, particularly asymptomatic left ventricular systolic dysfunction, and congestive heart failure.^3^, ^14^, ^15^, ^45^ The risk of cardiotoxicity can vary depending on multiple factors, including the specific type of anthracycline used, the total dose of anthracycline administered, the patient's age at the initiation of treatment, and the presence of other risk factors for heart damage such as mediastinal irradiation, preexisting heart conditions, and simultaneous exposure to other cardiotoxic agents, such as trastuzumab.^18^ This problem is particularly concerning for breast cancer survivors because it can affect their quality of life and increase their long‐term non‐breast cancer‐related mortality risk. To mitigate the risk of cardiotoxicity, patients receiving trastuzumab should undergo baseline cardiac assessments and regular monitoring during treatment, especially those who have previously received anthracyclines. The current recommended maximum cumulative doses of doxorubicin (<450 mg/m^2^),^22^ and epirubicin (<900 mg/m^2^) were determined through univariate analysis, using Kaplan–Meier estimates of cardiotoxicity as a function of cumulative dose.^11^, ^13^, ^15^, ^46^ However, the maximum cumulative doses of anthracyclines in concurrent use with trastuzumab remain undetermined. In the pivotal trastuzumab trial H0648g, 143 patients with metastatic HER2‐positive breast cancer received the trastuzumab/anthracycline combination and the incidence of symptomatic heart failure in the trastuzumab/anthracycline arm was 27%, with majority of patients receiving doxorubicin.^3^ In a multicenter prospective phase I/II trial, HERCULES,^47^ which evaluates epirubicin/cyclophosphamide plus trastuzumab as front‐line treatment for HER2‐positive metastatic breast cancer, patients were randomized to receive HEC‐90 or HEC‐60: trastuzumab administered concurrently with cyclophosphamide and epirubicin at either 60 mg/m^2^ (HEC‐60) or 90 mg/m^2^ (HEC‐90). The triweekly chemotherapy was prescribed for only 6 cycles while trastuzumab maintenance therapy could be given until disease progression. The primary endpoint of HERCULES was dose‐limiting cardiotoxicity (DLC) rate, which was defined as symptomatic heart failure associated with an absolute decrease in LVEF of more than 10 percentage points to less than 50%. The DLC rate of HEC‐90 was 5%, which is much less compared to H0648g. With enhanced understanding of anthracycline‐related cardiotoxicity, proactive monitoring, timely diagnosis, and appropriate management have been determined to be effective in reducing the risk of ACE during and after anthracycline treatment. Recently, the global longitudinal strain measured by echocardiography emerged as a valuable tool for the early diagnosis of chemotherapy‐induced cardiotoxicity.^20^

The meta‐analytical study constituted the most comprehensive evaluation of cardiotoxicity between concurrent epirubicin–trastuzumab and nonconcurrent regimens to date, comprising 1797 patients with HER2‐positive early breast cancer in seven studies. Adverse cardiac events were reported in 96 of these patients, with 6% occurring in the concurrent regimen arm (54 out of 899) and 4.7% occurring in the nonconcurrent regimen arm (42 out of 898). The median epirubicin dose was 300 mg/m^2^, ranging from 270 to 360 mg/m^2^, representing approximately one‐third of the recommended maximum cumulative dose. No significant differences in cardiotoxicity were observed between the concurrent and nonconcurrent regimens, even with two comparisons of non‐anthracycline regimens, which were previously thought to be much less cardiotoxic and might bring significant heterogeneity to our meta‐analysis. Examining the potential clinical and methodological reasons for the observed heterogeneity among the included trials provided deeper insights into the nuances of the study findings and the applicability of the results in different patient populations. In this study, we reported the original heterogeneity among the trials in the forest plots of Figures 2 and 3, but we found that the residual heterogeneity was not statistically significant after removing the effects of covariates in the corresponding linear fixed‐effects meta‐regression models in Table 2A,B. In our meta‐regression analyses of cardiotoxicity and pCR, we added all the available sources of heterogeneity to the “variable list” to be selected during the modern stepwise variable selection procedure (with iterations between the forward and backward steps). In particular, we purposely specified a dummy variable “Control_no.EorA” to accommodate the two studies with non‐anthracycline regimens as controls, but it did not stay in the final linear fixed‐effects meta‐regression models (Table 2A,B).

Furthermore, a significantly higher pCR rate was observed with the concurrent regimen compared to the nonconcurrent regimen. This finding remained consistent in the era of dual HER2‐blockade, whether using pertuzumab or lapatinib. These results suggest that the concurrent addition of epirubicin to trastuzumab therapy increases the pCR rate without an accompanying increase in cardiotoxicity. In our subsequent multivariate analysis, we found that in trials with a higher proportion of patients with HR‐positive breast cancer, the increase in the pCR rate was less significant. Compared to HR‐positive, HER2‐positive breast tumors, HR‐negative, HER2‐positive tumors are more likely to achieve pCR.^48^, ^49^ According to our study, shifting to concurrent regimens did not increase the likelihood of achieving pCR in HR‐positive, HER2‐positive tumors. Furthermore, cardiotoxicity increased in patients with HR‐positive, HER2‐positive breast cancer who received concurrent regimens. Therefore, based on our findings, the use of concurrent regimens for HR‐negative, HER2‐positive tumors to potentially improve the pCR rates could be considered. However, caution is advised when applying these regimens to HR‐positive, HER2‐positive tumors.

The reason for increased cardiotoxicity in patients with HR‐positive, HER2‐positive breast cancer receiving concurrent regimens remains unknown. Compared to patients with HR‐negative, HER2‐positive tumors, those with HR‐positive, HER2‐positive tumors were generally older. Additionally, most patients with early HR‐positive breast cancer receive endocrine therapy, which might also contribute to the observed cardiotoxicity. Further research is needed to establish any causal relationship between these factors.

This study had some limitations that should be addressed. First, the exact rate of cardiotoxicity could not be determined due to variations in the definition of cardiotoxicity in the included studies. Second, the mandatory schedule for echocardiography follow‐up differed among the 7 trials, and only one study (TRYPHAENA) provided follow‐up data at the 2‐year mark. Thus, ACE that occurred after 2 years without clinical symptoms might not have been detected.

Several anti‐HER2 targeted agents have recently been approved, including trastuzumab deruxtecan and tucatinib, for the treatment of metastatic HER2‐positive breast cancer. However, in cases where the disease progresses after such treatments, options may be limited to a combination of trastuzumab and chemotherapy. Thus, the cardiac safety of this treatment approach should be considered, particularly for patients who have received prior anti‐HER2 therapy. We hope that the current study provided valuable evidence on the cardiac safety of this combination therapy.

## 5 CONCLUSIONS

The concurrent administration of epirubicin and trastuzumab, with a limited dose of epirubicin, demonstrated a substantial positive impact on the pCR rate without an associated increase in cardiotoxicity. We recommend exploring the application of concurrent regimens for HR‐negative, HER2‐positive tumors as these may enhance pCR rates. Nevertheless, it is imperative to exercise caution when considering these regimens for HR‐positive, HER2‐positive tumors due to potential cardiotoxicity.

## AUTHOR CONTRIBUTIONS

**Ming‐Han Yang:** Data curation (lead); formal analysis (lead); investigation (lead); project administration (lead); writing – original draft (lead). **Chiun‐Sheng Huang:** Supervision (supporting); writing – review and editing (supporting). **Dwan‐Ying Chang:** Validation (equal); writing – review and editing (equal). **Fu‐Chang Hu:** Data curation (lead); formal analysis (lead); methodology (lead); software (lead); writing – original draft (supporting). **Shu‐Min Huang:** Funding acquisition (supporting); resources (supporting); supervision (supporting); validation (supporting). **Po‐Hsiang Huang:** Writing – review and editing (supporting). **I‐Chun Chen:** Validation (supporting); writing – review and editing (supporting). **Tom Wei‐Wu Chen:** Validation (supporting); writing – review and editing (supporting). **Ching‐Hung Lin:** Supervision (supporting); validation (supporting); writing – review and editing (supporting). **Yen‐Shen Lu:** Conceptualization (lead); formal analysis (equal); funding acquisition (lead); investigation (equal); methodology (lead); supervision (lead); validation (lead); writing – review and editing (lead).

## FUNDING INFORMATION

NSTC 111‐2314‐B‐002‐042 and MOHW 112‐TDU‐B‐211‐144002.

## CONFLICT OF INTEREST STATEMENT

Dr. Lu reports consulting and speaker fees from Novartis, Pfizer, Roche, and Merck Sharp & Dohme and study grants from Novartis, Roche, and Merck Sharp & Dohme. The other authors have no related conflicts of interest to disclose.

## CONSENT FOR PUBLICATION

All authors approved the manuscript submitted for publication.

## Supporting information


Appendix S1:

Appendix S2:

## Data Availability

The datasets were built from those selected trials and please refer to the referenced trials.
