# Supplementary material for: Concurrent epirubicin and trastuzumab use increases complete pathological response rate without additional cardiotoxicity in patients with human epidermal growth factor receptor 2‐positive early breast cancer: A meta‐regression analysis
Source: Cancer Med. 2024 Jul 24;13(14):e70005. doi: 10.1002/cam4.70005 (PMC11267450; doi:10.1002/cam4.70005)
Supplement: Supplementary file 1 — Appendix S1: [file CAM4-13-e70005-s001.docx]

**Supplementary Appendix 1**

**Contents**

[**Table S1. Literature Search Strategies………………………………………………………………………P. 2**](#TableS1)

[**Table S2. Risk of Bias Assessments……………………………………………….………………………… P. 3**](#TableS2)

[**Table S3. Definition of Cardiotoxicity……………………………………...…….………………………… P. 4**](#TableS3)

[**Figure S1. GAM Plots – Average HR+ Participants vs. Cardiotoxicity / pCR Rate…...............................P. 5**](#FigureS1)

[**Figure S2. Funnel Plots of Fixed-Effects Meta-Regression Models..............................................................P.**](#FigureS2) **7**

Table S1. Literature Search Strategies

| **Database** | **Search Strategy** | **Results** | **Date** |
| --- | --- | --- | --- |
| Pubmed | “HER2 positive breast cancer” AND “neoadjuvant” OR “adjuvant” AND “trastuzumab” AND “anthracycline" AND clinical trial[Filter] OR randomized controlled trial[Filter] | 178 | 2021/02/28 |
| Cochrane Library | Title, abstract, and keywords: “HER2 positive breast cancer” AND “trastuzumab” AND “anthracycline"  Search limits: trials (content type); otherwise: default | 112 | 2021/02/28 |

**Table S2. Risk of Bias Assessments**

|  |  |  | **Domain**^*^ | | | | |  |
| --- | --- | --- | --- | --- | --- | --- | --- | --- |
| **Trial**  **Name** | **Experimental**  **Treatment** | **Control**  **Treatment** | **1** | **2** | **3** | **4** | **5** | **Overall Bias** |
| Buzdar MDACC 2005 | TH (4) 🡪 FECH (4) | T (4) 🡪 FEC (4) | Low | Low | Low | Low | Low | Low |
| CHER-LOB | wTH (12) 🡪 FECH (4) wTHL (12) 🡪 FECHL (4) | wTL (12) 🡪 FECL (4) | Low | Low | Low | Low | Low | Low |
| GeparQuinto GBG 44 | ECH (4) 🡪 DH (4) | ECL (4) 🡪 DL (4) | Low | Low | Low | Low | Low | Low |
| TRYPHAENA | FECPH (4) 🡪 DPH (4) | FEC (4) 🡪 DPH (4) DCarboH (6) | Low | Low | Low | Low | Low | Low |
| ACOSOG Z1041 | TH 🡪 FECH | FEC 🡪 TH | Low | Low | Low | Low | Low | Low |
| EORTC 10054 | DH 🡪 FECH | DL 🡪 FECL | Low | Low | Low | Some Concerns^**^ | Low | Low |
| TRAIN-2 | FECPH 🡪 TCPH | TCPH | Low | Low | Low | Low | Low | Low |

**Abbreviations:** T(H) paclitaxel (+ Herceptin), FEC(H) fluorouracil + epirubicin + cyclophosphamide (+ Herceptin), D(H) docetaxel (+ Herceptin), TLH paclitaxel + lapatinib + Herceptin, FECPH fluorouracil + epirubicin + cyclophosphamide + pertuzumab + Herceptin, DPH docetaxel + pertuzumab + Herceptin, DCarboPH docetaxel + carboplatin + pertuzumab + Herceptin, TCarboPH paclitaxel + carboplatin + pertuzumab + Herceptin

^*^ The domains of risk-of-bias assessment include (1) randomization process, (2) deviations from intended interventions, (3) missing outcome data, (4) measurement of the outcome, and (5) selection of reported result.

^**^ EORTC 10054 reported cardiotoxicity, but the number of events was zero in both groups.

**Table S3. Definition of Cardiotoxicity**

| Item | Definition | Gr 1 | 2 | 3 | 4 | 5 |
| --- | --- | --- | --- | --- | --- | --- |
| LVEF decrease (non-CTCAE) | LVEF decline >= 10% **AND** LVEF< 50% | - | - | - | - | - |
| LVSD  (CTCAE 3.0) | Left ventricular systolic dysfunction | Asymptomatic, resting EF <60-50%; shortening fraction (SF) <30 -24% | Asymptomatic, resting EF <50–40%; SF <24–15% | **Symptomatic CHF** responsive to intervention; EF <40 – 20% SF <15% | **Refractory CHF** or poorly controlled; EF <20%; intervention such as VAD, surgery, or heart transplant indicated | Death |
| Heart failure (CTCAE 4.03) |  | Asymptomatic with BNP or cardiac imaging abnormalities | Symptoms with mild to moderate activity or exertion | Severe with symptoms at rest or with minimal activity or exertion; intervention indicated | Life-threatening consequences; urgent intervention indicated (e.g., continuous IV therapy or mechanical hemodynamic support) | Death |
| LVSD  (CTCAE 4.03) | Left ventricular systolic dysfunction | - | - | Symptomatic due to drop in EF responsive to intervention | Refractory or poorly controlled heart failure due to drop in EF; intervention such as VAD, IV vasopressor support, or heart transplant indicated | Death |
| LVEF decreased (CTCAE 4.03) |  | - | EF: 50%-40% or 10-19% drop from baseline | EF: 39%-20% or >20% drop | EF<20% | - |
| NYHA Fc | Functional class | No limitation of physical activity. | Slight limitation of physical activity. | Marked limitation of physical activity. | Unable to carry on any physical activity without discomfort. | - |

**Figure S1A. GAM Plot — Average Percentage of HR+ Participants vs. Predicted Value of log(RR) of Cardiotoxicity**


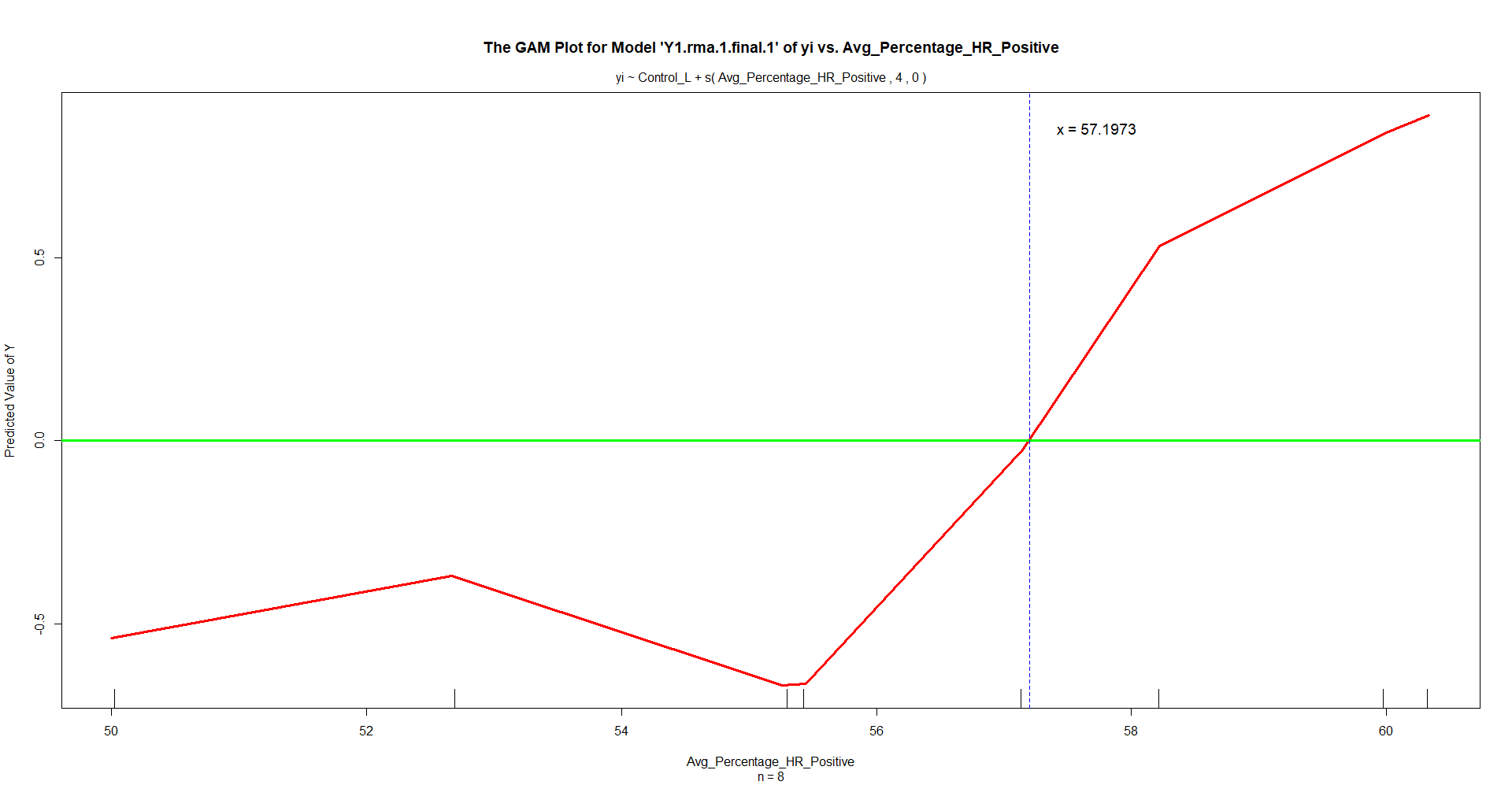


**Figure S1B. GAM Plot — Average Percentage of HR+ Participants vs. Predicted Value of log(OR) of pCR Rate**


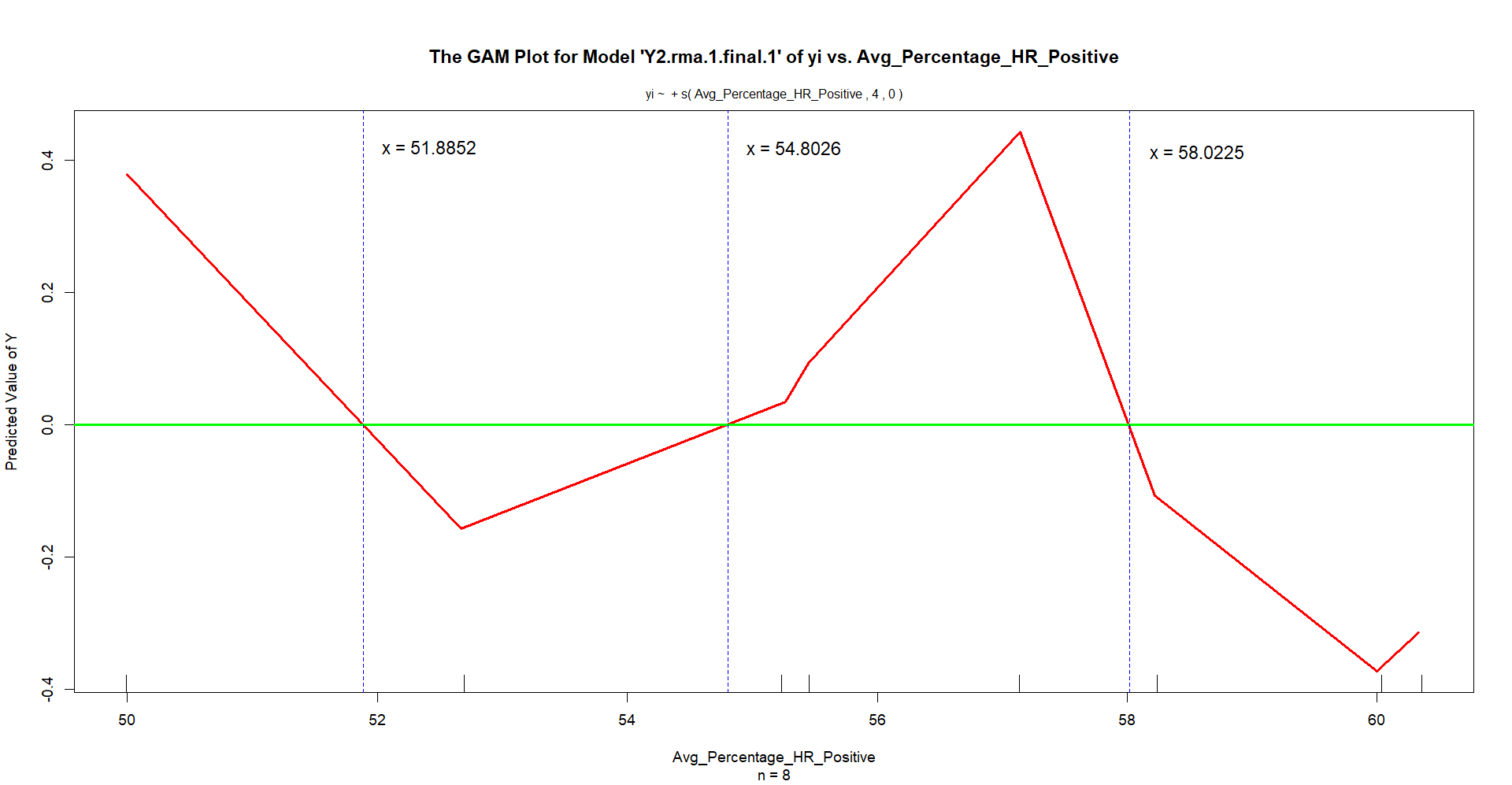


**Figure S2A. The Funnel Plot of the Fixed-Effects Meta-Regression Model for log(RR) of Cardiotoxicity**

**
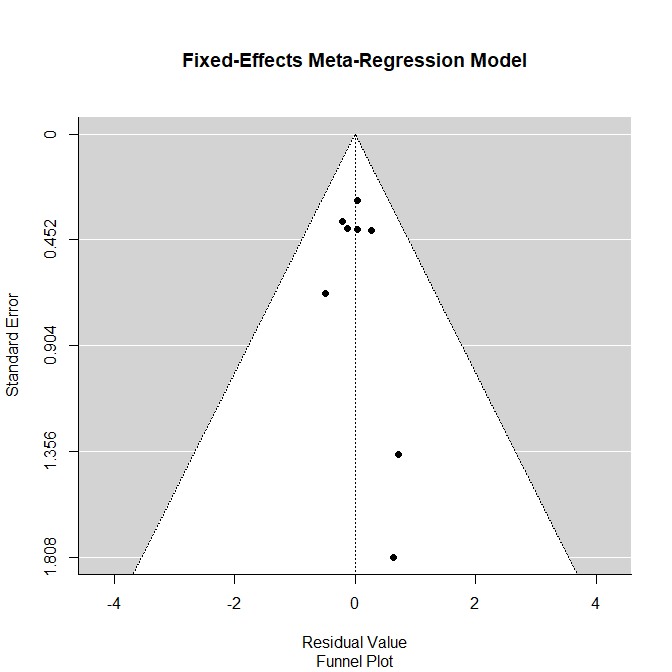
**

**Figure S2B. The Funnel Plot of the Fixed-Effects Meta-Regression Model for log(OR) of the pCR Rate**

**
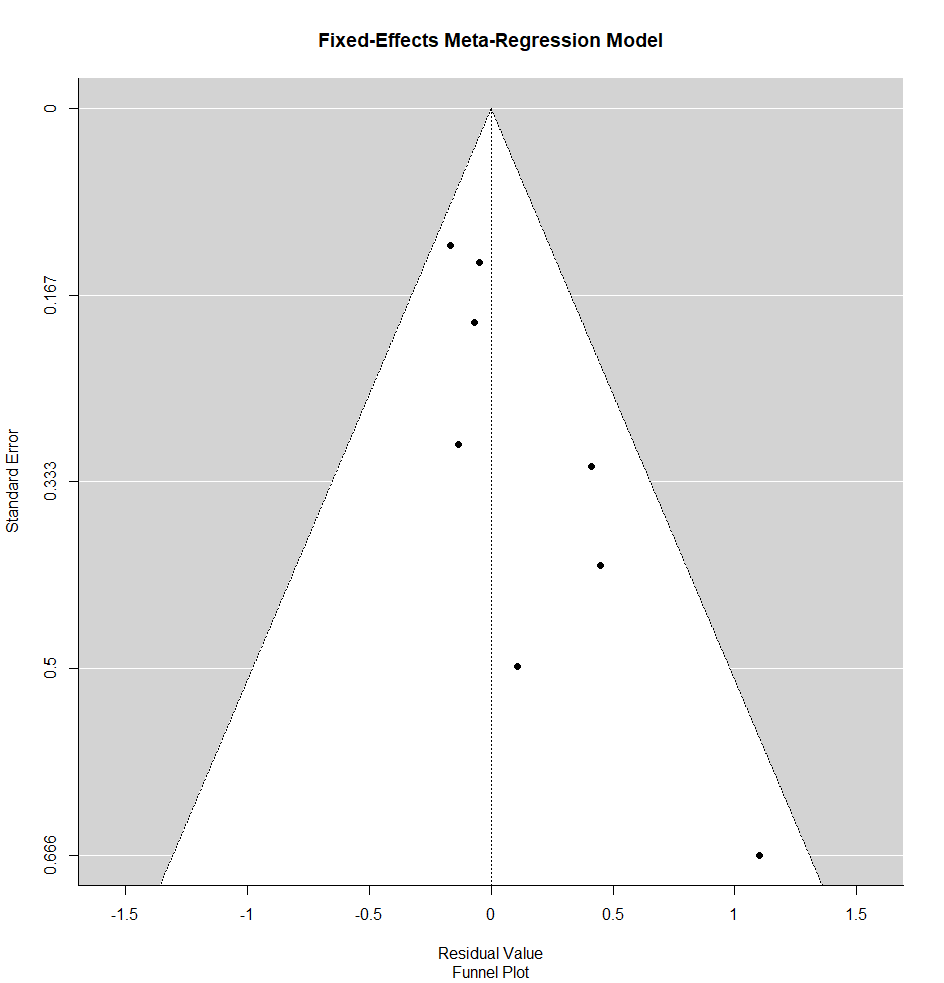
**
