# Supplementary material for: Concurrent epirubicin and trastuzumab use increases complete pathological response rate without additional cardiotoxicity in patients with human epidermal growth factor receptor 2‐positive early breast cancer: A meta‐regression analysis
Source: Cancer Med. 2024 Jul 24;13(14):e70005. doi: 10.1002/cam4.70005 (PMC11267450; doi:10.1002/cam4.70005)
Supplement: Supplementary file 2 — Appendix S2: [file CAM4-13-e70005-s002.docx]

**Supplementary Appendix 2**

**The Smoothed-Effect Plots**

***Linear regression analysis***

In our linear regression analysis, simple and multiple *generalized additive models* (GAMs) were fitted to detect the nonlinear effects of continuous covariates and identify the appropriate cut-off point(s) for discretizing continuous covariates, if necessary, during the modern stepwise variable selection procedure. Computationally, the vgam() function (with the default values of the smoothing parameters) of the VGAM package (Yee and Wild, 1996; Yee, 2015, 2018) was used to fit the GAMs for continuous responses in R. According to the fitted GAM of a continuous outcome variable, the curve drawn in the GAM plot helps us visualize the effect of a continuous covariate (*X*-axis) on the predictive value of the continuous outcome variable, $\hat{P}$ (*Y*-axis). The short vertical bars (called the ‘rug’) above the *X*-axis indicated where the observed values were located. In such a GAM plot, the intersection(s) between the horizontal green line and the red curve yields the estimated optimal cut-off value(s) of the *X*-axis, at which $\hat{P}$ will *not* increase or decrease. When the covariate values > an estimated optimal cut-off value, the value of $\hat{P}$ increases or decreases, depending on how the red curve goes, and *vice versa*. In other words, the *Y*-axis of this GAM plot is the rise or decline in the value of $\hat{P}$ (i.e., Δ $\hat{P}$) with the horizontal green line at Δ $\hat{P}$ = 0.

***Logistic regression analysis***

In our logistic regression analysis, simple and multiple *generalized additive models* (GAMs) were fitted to detect the nonlinear effects of continuous covariates and identify the appropriate cut-off point(s) for discretizing continuous covariates, if necessary, during the modern stepwise variable selection procedure. Computationally, the vgam() function (with the default values of the smoothing parameters) of the VGAM package (Yee and Wild, 1996; Yee, 2015, 2018) was used to fit the GAMs for binary responses in R. According to the fitted GAM of a binary outcome variable, the curve drawn in the GAM plot helps us visualize the effect of a continuous covariate (*X*-axis) on logit(*P*) (*Y*-axis), where logit(*P*) = log(*P*/(1−*P*)) and *P* is the probability of having the outcome event. The short vertical bars (called the ‘rug’) above the *X*-axis indicated where the observed values were located. In such a GAM plot, the intersection(s) between the horizontal green line and the red curve yields the estimated optimal cut-off value(s) of the *X*-axis, at which the value of logit(*P*) will *not* increase or decrease. When the covariate values > an estimated optimal cut-off value, the value of logit(*P*) increases or decreases, and then *P* increases or decreases accordingly, depending on how the red curve goes, and *vice versa*. In other words, the *Y*-axis of this GAM plot is the rise or decline in the value on the logit(*P*) scale (i.e., Δ logit(*P*)) with the horizontal green line at Δ logit(*P*) = 0.

***Cox’s proportional hazards model***

In our regression analysis, smoothing techniques were applied to detect the nonlinear effects of continuous covariates and identify the appropriate cut-off point(s) for discretizing continuous covariates, if necessary, during the modern stepwise variable selection procedure. Computationally, we speciﬁed the smoothing option pspline (for the smoothing splines using a “p-spline” basis) inside the coxph() function of the survival package to smooth the eﬀects of continuous covariates on the survival outcome of simple and multiple Cox’s proportional hazards models in R ― for example, specified the pspline(age, df=4) term inside the coxph() function for age, and then used the termplot() function of the stats package to plot the smoothed eﬀect of age on the log-hazard rate (Moore, 2016, pp. 84-85). As a result, the curve drawn in a p-spline plot helps us visualize the effect of a continuous covariate (*X*-axis) on log(*λ*) (*Y*-axis), where *λ* is the hazard rate of having the outcome event. The short vertical bars (called the ‘rug’) above the *X*-axis indicated where the observed values were located. In such a p-spline plot, the intersection(s) between the horizontal green line and the red curve yields the estimated optimal cut-off value(s) of the *X*-axis, at which the value of log(*λ*) will *not* increase or decrease. When the covariate values > an estimated optimal cut-off value, the value of log(*λ*) increases or decreases, and then *λ* increases or decreases accordingly, depending on how the red curve goes, and *vice versa*. In other words, the *Y*-axis of this p-spline plot is the rise or decline in the value on the log(*λ*) scale (i.e., Δ log(*λ*)) with the horizontal green line at Δ log(*λ*) = 0.

**References**

Moore, D. F. (2016). *Applied Survival Analysis Using R*. Cham: Springer International Publishing Switzerland.

Yee, T. W. (2015). *Vector Generalized Linear and Additive Models: With an Implementation in R*. New York, NY: Springer Science+Business Media.

Yee, T. W. (2018). VGAM: Vector generalized linear and additive models. R package, version 1.0-6 (URL: <https://CRAN.R-project.org/package=VGAM>).

Yee, T. W. and Wild, C. J. (1996). Vector generalized additive models. *Journal of Royal Statistical Society*, Series B, 58(3): 481−493.
